# *Radix pseudostellariae* Saponins Promote Immunocyte Migration and Chemotaxis via the CCL5/CCR4 Signaling Axis

**DOI:** 10.3390/ani16121929

**Published:** 2026-06-22

**Authors:** Jiaqi Chen, Xiangduan Wei, Yuting Cao, Beilei Chen, Qixian Feng, Zhengrun Xiao, Lihui Xu, Yufang Ma, Quanxi Wang

**Affiliations:** 1 Fujian Key Laboratory of Traditional Chinese Veterinary Medicine and Animal Health, College of Animal Science, Fujian Agriculture and Forestry University, Fuzhou 350002, China; 2 University Key Laboratory for Integrated Chinese Traditional and Western Veterinary Medicine and Animal Healthcare in Fujian Province, College of Animal Science, Fujian Agriculture and Forestry University, Fuzhou 350002, China

**Keywords:** *Radix pseudostellariae* saponins, CCL5/CCR4 signaling axis, immune cell migration, synergistic immune enhancement

## Abstract

*Radix pseudostellariae* saponins (RPS) enhance immune responses in animals; however, the regulatory mechanisms of these effects remain unclear. This study observed that 14 days post-intranasal immunization with RPS and a *Mycoplasma gallisepticum*-attenuated vaccine (MGAV), MGAV-specific antibody titers were significantly increased in the blood, and *chemokine* (C-C motif) *ligand 5* (CCL5) messenger RNA expression was significantly increased in the trachea and blood of chickens. Transcriptomic analysis demonstrated that RPS treatment significantly upregulated specific Kyoto Encyclopedia of Genes and Genomes pathways, notably the cytokine–cytokine receptor interaction pathway, which is linked to immune cell migration and involves *chemokine receptor chemokine* (C-C motif) *receptor* 4 (CCR4). This finding was corroborated at the protein level by immunohistochemical evidence showing increased CCL5 expression in tracheal tissue. In vitro studies showed that RPS enhanced the phagocytic capacity of RAW264.7 macrophages against *ovalbumin*, with *immunofluorescence* revealing time-dependent and dose-dependent CCL5 in these cells. Transwell and scratch-healing assays confirmed that RPS promoted this migration of both RAW264.7 cells and CCR4-positive lymphocytes. Collectively, the findings revealed that RPS modulated the activation, chemotaxis, and migration of macrophages and lymphocytes and is associated with the promotion of the CCL5/CCR4 signaling axis, providing novel evidence for the immune-enhancing effects of RPS by enhancing immunogenicity.

## Simple Summary

This study is the first to elucidate the immune-enhancing mechanism of *Radix pseudostellariae* saponins when used in conjunction with vaccines. Using a combination of in vivo and in vitro approaches, this study provided multidimensional validation of the immunomodulatory functions mediated by the chemokine C-C motif ligand 5/chemokine (C-C motif) receptor 4 signaling axis. This study demonstrated that when administered intranasally alongside a *Mycoplasma gallisepticum*-attenuated vaccine, *Radix pseudostellariae* saponins significantly increased vaccine-specific antibody titers and upregulated key immune-related genes, including *chemokine C-C motif ligand 5* and *chemokine* (C-C motif) *receptor 4*, in both mucosal and systemic tissues. Transcriptomic profiling, immunohistochemistry, and functional cellular assays confirmed that *Radix pseudostellariae* saponins promoted macrophage phagocytosis and enhanced antigen presentation. It also facilitated the migration of *chemokine* (C-C motif) *receptor 4*-positive lymphocytes. All of these effects were orchestrated through *chemokine C-C motif ligand 5*/*chemokine* (C-C motif) *receptor 4* pathway activation. The findings reveal a novel molecular mechanism underlying the candidate adjuvant activity of *Radix pseudostellariae* saponins and offer compelling scientific evidence supporting the use of *Radix pseudostellariae* saponins as a traditional Chinese medicine-derived immunomodulator in vaccine formulations.

## 1. Introduction

*Mycoplasma gallisepticum* (MG) is a globally prevalent avian pathogen characterized by complex epidemiological dynamics and a broad host range. Primarily invading the respiratory system, a recent study indicated that MG frequently co-infects with bacteria or viruses, exacerbating disease severity and economic losses [1]. Current MG vaccines exhibit multiple limitations in terms of efficacy, safety, differentiation from field strains, and antibiotic compatibility [2,3]. Inactivated bacterial vaccines, while inducing strong systemic immunoglobulin (Ig)G antibody responses, fail to elicit effective mucosal immunity, thereby limiting their ability to prevent MG respiratory colonization. Live vaccines (e.g., serogroup F, TS-11, and 6/85 strains) can colonize the upper respiratory tract and effectively prevent clinical symptoms, but they cannot completely block transmission or reinfection [4,5,6,7]. The wing-vein injection technique used in certain Avian Encephalomyelitis -MG combination vaccines has been linked to vaccine-derived neuropathogenic outbreaks, highlighting the risk of unintended pathogenicity [8]. Furthermore, reduced vaccine efficacy under antibiotic conditions undermines the effectiveness of integrated disease management strategies combining vaccination with drug therapy [9,10].

*Radix pseudostellariae* (RP), also known as Taizisen, is a traditional Chinese medicine, which is believed to “tonify qi” in traditional Chinese medicine, a concept distinct from measurable biological effects, as well as to promote fluid production, strengthen the spleen, and nourish the lungs [11]. It has been shown that RP saponins (RPS) enhance immune function, as well as exerting anti-inflammatory and anti-tumor effects [12]. Chen et al. revealed that feeding RPS to mice immunized with *ovalbumin* partially protected the intestinal tissue morphology and regulated Secretory IgA and related cytokine secretion, thereby enhancing intestinal mucosal immunity and improving the immune response of mice to ovalbumin [13]. These findings indicate that RPS possesses certain immunostimulatory effects and functions as a vaccine adjuvant.

*Chemokine C-C motif ligand 5* (CCL5), also known as Regulated upon Activation, Normal T-cell Expressed and Secreted (RANTES), is a multifunctional chemokine that plays a crucial role in immune regulation, inflammation, and tumor microenvironment remodeling [14,15]. CCL5 is expressed in monocytes and T cells [16], and it exerts concentration-dependent effects on T cells, inducing migration at low concentrations and promoting activation at high concentrations [17]. While chemokine receptor CCR5 (C-C motif receptor 5) is its primary receptor, mediating inflammatory and defense responses, CCL5 also binds *chemokine receptor chemokine* (C-C motif) *receptor* 4 (CCR4) [18,19]. CCR4 has been identified as a regulator of regulatory T cell (Treg)/T helper 2 (Th2) cell function and is considered a potential immunomodulatory target [20,21]. Although the role of CCR4 in recruiting cells to tumors and its activation via CCL17/ERK/STAT3 in Th2 cells [22] are well-established, the role of CCL5/CCR4 in specific microenvironments, such as RPS-enhanced immunity, remains unclear. Elucidating these mechanisms is crucial for two purposes: (1) to validate the scientific basis of RPS as an immunomodulator and (2) to optimize its application in vaccine formulations and develop novel adjuvant strategies based on natural products.

The precise molecular mechanism remains unclear, particularly in terms of the initiation of signals and the specific interactions between chemokines and immunocytes upon RPS stimulation. Given the conservation of chemokine signaling pathways between avian and mammalian species, particularly the CCL5/CCR4 axis, RAW264.7 murine macrophages were used as a mechanistic proxy to enable detailed molecular dissection, as mature commercial chicken macrophage cell lines are lacking/unavailable. In this study, we hypothesized that RPS would enhance immunity by promoting the migration of immune cells through the CCL5/CCR4 axis in chickens. First, the immunostimulatory effect of RPS was demonstrated in chickens by intranasal immunization. Subsequent transcriptomic and quantitative real-time polymerase chain reaction (qPCR) analyses revealed that the CCL5/CCR4 pathway was activated. Finally, in vitro experiments were performed to confirm that RPS regulated macrophage chemotaxis and recruited CCR4-positive cells via the CCL5/CCR4 signaling axis.

## 2. Materials and Methods

### 2.1. Animals and Cells

Thirty-day-old chickens were obtained from Hetian Chicken Breeding Farm (Changting County, Fujian, China). Mice from the specific pathogen-free Institute of Cancer Research (ICR) were purchased from Fuzhou Wushi Experimental Animal Company (Fuzhou, China). RPS (73% total saponins) was purchased from Waltlet Biotechnology Co., Ltd. (Lanzhou, China). RAW264.7 macrophages were preserved in our laboratory.

### 2.2. Grouping and Immunization

Fifty-four MG-negative chickens were acclimated for 3 days before being divided into three groups (*n* = 18 per group), as follows: control group, 0.1 mL saline (intranasal); MGAV group, 3.0 × 10^6^ Color Change Unit /bird (Zhaofenghua Biotechnology [Fuzhou] Co., Ltd., Fuzhou, China); and MGAV + RPS group, MGAV + 20 mg·kg^−1^ RPS. Boosters at day 28. Six birds per group sampled at day 42 for serum/spleen/trachea.

### 2.3. qPCR Analysis

Total RNA from each of the trachea, blood, and spleen was extracted and reverse transcribed into complementary DNA (*ER501-01-V2*, TransGen Biotech [Beijing] Co., Ltd., Beijing, China). The relative expression of *interleukin-4* (IL-4), *CCL5*, and *CCR4* was evaluated by qPCR, using *β-actin* as the internal reference gene [23]. The specific primers are specified in Table A1.

### 2.4. Immunohistochemistry

Tracheal samples were fixed in 4% paraformaldehyde for >24 h, followed by dehydration, clearing, and paraffin embedding. The tissues were sectioned to a thickness of 4–6 μm. After deparaffinization and rehydration, the sections were subjected to heat-induced antigen retrieval (100 °C, 25 min). Endogenous peroxidase activity was blocked using 3% hydrogen peroxide for 10 min, followed by washing in phosphate-buffered saline (PBS) and blocking with 3% bovine serum albumin (BSA) for 10 min. The sections were then incubated with rabbit anti-CCL5/RANTES polyclonal antibody (ImmunoWay, Suzhou, China) at a 1:2000 dilution for 20 min at 37 °C. After PBS washing, the sections were incubated with horseradish peroxidase-conjugated secondary antibody (Servicebio, Wuhan, China) for 20 min at room temperature. The reaction was visualized using Diaminobenzidine (DAB) substrate for 5 min, and the reaction was stopped by rinsing with tap water. The sections were counterstained using hematoxylin for 10 min, followed by a bluing step under running tap water. After gradient ethanol dehydration (70%, 80%, 90%, 95%, and 100% ethanol, sequentially), clearing in xylene, and mounting with neutral balsam, the sections were observed and photographed by light microscopy. A brownish-yellow granular deposit in the cytoplasm or cell membrane was considered a positive signal. The H-score for CCL5 staining was calculated using the formula Σ(pi × i), where pi represents the percentage of positive cells corresponding to each intensity grade (0: no staining, 1: weak, 2: moderate, 3: strong), yielding a score from 0 to 300.

### 2.5. Transcriptomic Screening for Differentially Expressed Genes (DEGs)

Spleen samples were collected and sent to Shanghai Ouyi Biotechnology Co., Ltd. (Shanghai, China) for sequencing on the Illumina NovaSeq 6000 platform (Illumina, San Diego, CA, USA), generating 150 base pair paired-end reads. After quality control using fastp software 0.22.0 (Available online: https://github.com/OpenGene/fastp, accessed on 1 January 2022; OpenGene), the clean reads were aligned to the reference genome using HISAT2 software V2.2.1 (Available online: https://ccb.jhu.edu/software/hisat2/index.shtml, accessed on 6 November 2022; Johns Hopkins University, Baltimore, MD, USA). Gene read counts were obtained using HTSeq-count (Available online: https://htseq.readthedocs.io, accessed on 17 February 2022; Python package 3.10.4). *Principal component analysis* for assessing biological replicates was performed using the prcomp function of R software (version 3.2.0; R Foundation for Statistical Computing, Vienna, Austria). DEGs were identified using the DESeq2 package (version 1.24.0; Available online: https://bioconductor.org/packages/DESeq2, accessed on 16 March 2022; Bioconductor) with thresholds of adjusted *p*-value (FDR) < 0.05 and |fold change| > 2. Hierarchical clustering of DEGs was conducted using the pheatmap package (Available online: https://cran.r-project.org/web/packages/pheatmap, accessed on 24 October 2010) of R software. Functional enrichment analyses, including *Gene Ontology* (GO) and *Kyoto Encyclopedia of Genes and Genomes* (KEGG) pathway analyses, were performed using the clusterProfiler package (Available online: https://bioconductor.org/packages/clusterProfiler, accessed on 27 October 2021; Bioconductor) of R software. *Gene Set Enrichment Analysis* (GSEA) was performed using GSEA software (version 4.1.0; Broad Institute, Cambridge, MA, USA; Available online: https://www.gsea-msigdb.org/gsea, accessed on 3 October 2022) to evaluate the enrichment levels of predefined gene sets within the expression profiles. The sequencing data were validated by qPCR.

### 2.6. In Vitro Assays

For immunofluorescence detection of phagocytosis, RAW264.7 cells were treated with 200 μg·mL^−1^ ovalbumin, 200 μg·mL^−1^ RPS, or 200 μg·mL^−1^ ovalbumin + 100 μg·mL^−1^ RPS, separately, for 12 h. The cells were fixed using 4% paraformaldehyde, permeabilized using 0.1% Triton X-100, and incubated with anti-ovalbumin antibody (Cloud-Clone, Wuhan, China) and fluorescein isothiocyanate-conjugated goat anti-mouse IgG (Servicebio, Wuhan, China). The membranes were stained using the Dio Cell Membrane Green Fluorescence Staining Kit (Beyotime Biotechnology, Shanghai, China), mounted, and examined under a microscope [24].

RAW264.7 cells were seeded at a density of 5 × 10^5^ cells per well in 6-well plates containing 2 mL of DMEM medium. To evaluate the dose- and time-dependent expression of *CCL5*, RAW264.7 cells were treated with ovalbumin (0, 50, 100, 200, and 400 μg·mL^−1^) for 6 h, separately; or RPS (0, 25, 50, 100, and 200 μg·mL^−1^) for 6 h, separately. *CCL5* was determined by qPCR and *Western blotting* (WB). The optimum time for ovalbumin or RPS treatment was evaluated. The cells were treated with 100 μg·mL^−1^ ovalbumin or RPS for 0, 3, 6, or 12 h separately; *CCL5* was determined by qPCR and WB. Furthermore, the cells were subjected to combined treatment using 100 μg·mL^−1^ ovalbumin + 100 μg·mL^−1^ RPS, 100 μg·mL^−1^ ovalbumin + 200 μg·mL^−1^ RPS, or 200 μg·mL^−1^ ovalbumin + 100 μg·mL^−1^ RPS for 0, 3, 6, and 12 h, and *CCL5* was measured. RAW264.7 cells were treated with ovalbumin (0, 50, 100, 200, and 400 μg·mL^−1^) or RPS (0, 25, 50, 100, and 200 μg·mL^−1^) for 6 h. CCL5 expression was determined by qPCR and WB. To evaluate the optimal treatment time, the cells were treated with 100 μg·mL^−1^ ovalbumin or 100 μg·mL^−1^ RPS for 0, 3, 6, or 12 h, and CCL5 was again measured. For combined treatment, the cells were exposed to 100 μg·mL^−1^ ovalbumin + 100 μg·mL^−1^ RPS, 100 μg·mL^−1^ ovalbumin + 200 μg·mL^−1^ RPS, or 200 μg·mL^−1^ ovalbumin + 100 μg·mL^−1^ RPS for 0, 3, 6, or 12 h, and the mRNA levels expression of CCL5 was analyzed.

For silencing RNA (siRNA) transfection, RAW264.7 cells were seeded into 6-well plates for 18 h and transfected with 50 nM CCL5 siRNA or negative control using 1 μL/mL Lipo800™ transfection reagent (Beyotime Biotechnology) according to the manufacturer’s instructions.

For the Transwell migration assay, primary lymphocytes were isolated according to the Lymphocyte Isolation Kit protocol (Beijing Solarbio Science & Technology Co., Ltd., Beijing, China), removed to the upper chamber, and treated with PBS or CCR4 inhibitor. RAW264.7 cells were seeded in the lower chamber at a density of 1 × 10^5^ cells per well in 0.5 mL medium. Primary lymphocytes were seeded in the upper chamber at a density of 2 × 10^5^ cells per well in 0.1 mL medium. RAW264.7 cells were grown in the lower chamber and cultured with PBS, 100 μg·mL^−1^ ovalbumin + 200 μg·mL^−1^ RPS, or CCL5 siRNA + 100 μg·mL^−1^ ovalbumin + 200 μg·mL^−1^, separately. At 6 h post incubation, the migration ability of the cells was assessed, and the number of migrated cells was counted using Image J software 1.54p (National Institutes of Health, Bethesda, MD, USA).

For the scratch wound-healing assay, RAW264.7 cells were seeded into 6-well plates at a density of 5 × 10^5^ cells per well and cultured until confluent. After 24 h, vertical scratches were made, and PBS was replaced with serum-free medium. The cells were treated with PBS, 100 μg·mL^−1^ ovalbumin + 200 μg·mL^−1^ RPS, or CCL5 siRNA + 100 μg·mL^−1^ ovalbumin + 200 μg·mL^−1^ RPS. At 0, 6, 9, and 12 h post-treatment, the wound area was observed under an inverted microscope. The wound healing area was quantified using Image J software 1.54p, and the percentage wound closure was calculated as: (initial wound area − wound area at each time point) ÷ initial wound area × 100%. All experiments were performed in triplicate.

### 2.7. Statistical Analyses

All data were analyzed by one-way analysis of variance using GraphPad Prism 8 software (GraphPad Software, San Diego, CA, USA), and Tukey’s test was used for multiple comparisons. Significant differences are indicated by lowercase letters in the figures; the same letter indicates no significant difference among the groups (*p* > 0.05), while different letters indicate a significant difference (*p* < 0.05). All statistical results were plotted using GraphPad Prism 8 software.

## 3. Results

### 3.1. Combined Intranasal Administration of RPS and MGAV Enhanced MGAV Immunogenicity

**Figure 1:**
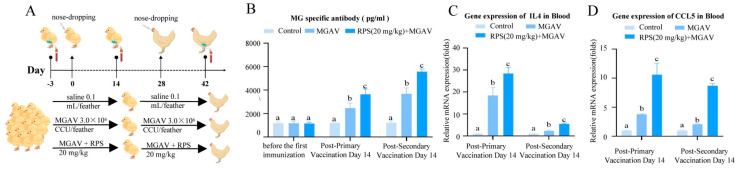
Nasal immunization of *Radix pseudostellariae* saponins (RPS) combined with *Mycoplasma gallisepticum*-attenuated vaccine (MGAV) enhanced the immune effect. (**A**) Fifty-four healthy 30-day-old MG-negative chickens were divided into three groups: (1) control group, intranasal administration of 0.1 mL phosphate-buffered saline (PBS) per bird; (2) MGAV group, intranasal administration of 3.0 × 10^6^ color change units MGAV per bird; and (3) combined group, intranasal co-administration of RPS (20 mg·kg^−1^) + MGAV. Twenty-eight days later, all groups received the booster vaccination. Peripheral blood samples were collected before vaccination, 14 days after the first vaccination, and 14 days after the second vaccination. (**B**) The concentrations of MG antibody were quantified by ELISA, and the mRNA expression of *interleukin-4 (IL-4)* (**C**) and *C-C motif ligand 5(CCL5*). (**D**) was measured by quantitative real-time polymerase chain reaction (qPCR). The data are presented as the mean ± SD and were analyzed by one-way ANOVA followed by Tukey’s post hoc test. In the figure, different lowercase letters indicate significant differences among the groups (*p* < 0.05), while the same letter indicates no significant difference among the groups (*p* < 0.05).

The immunization protocol involved primary vaccination on Day 0 and booster vaccination on Day 28 (Figure 1A). Serum samples were systematically collected at three timepoints: pre-vaccination baseline, 14 days post-primary vaccination, and 14 days post-booster vaccination. This longitudinal sampling strategy enabled comprehensive monitoring of humoral immune responses. As illustrated in Figure 1B, the MG-specific antibody titers were significantly elevated in the RPS + MGAV group at 14 days post-primary vaccination and post-booster vaccination compared with the MGAV-only group, indicating that co-treatment with RPS and the MGAV significantly enhanced immunogenicity.

Next, we evaluated the mRNA expression of the immune-related cytokines *IL-4* and *CCL5* across multiple tissues (trachea, blood, and spleen) by qPCR. Compared with the MGAV-only treatment, nasal immunization with RPS combined with the MGAV demonstrated a significant increase in *IL4* and *CCL5* mRNA expression in the blood (Figure 1C,D). These findings provide compelling evidence that RPS synergizes with the MGAV to amplify vaccine efficacy through coordinated modulation of both humoral and cellular immune pathways.

### 3.2. Screening and Validation of DEGs in the Spleen

**Figure 2:**
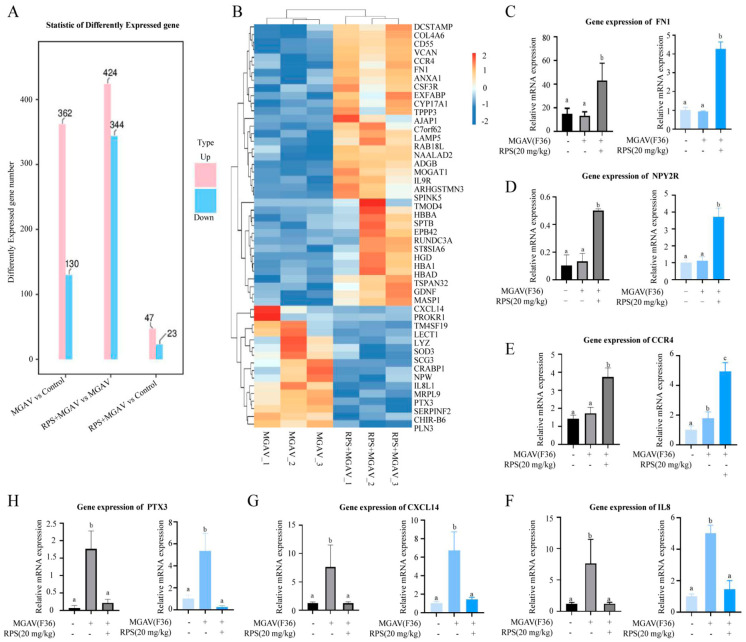
Transcriptome sequencing was used to screen DEGs in the spleen. Spleens from the control group, MGAV group, and RPS + MGAV group were sent for transcriptome sequencing. (**A**) DEGs were identified using DESeq2 and are presented as a heatmap (**B**). The mRNA expression of fibronectin 1 (FN1) (**C**), neuropeptide Y receptor type 2 (*NPY2R*) (**D**), chemokine C-C motif receptor 4 (*CCR4*) (**E**), C-X-C motif chemokine ligand 14 (*CXCL14*) (**F**), *interleukin-8* (*IL-8*) (**G**), and pentraxin 3 (*PTX3*) (**H**) was evaluated by qPCR. The trends observed between groups (left panel) in the transcriptome sequencing data are consistent with the trends observed within groups (right panel). In the figure, different lowercase letters indicate significant differences among the groups (*p* < 0.05), while the same letter indicates no significant difference among the groups (*p* < 0.05).

To investigate the immunomodulatory mechanism of RPS at the transcriptional level, we conducted comprehensive RNA sequencing of spleen tissues. Comparative transcriptomic profiling identified 424 DEGs (Figure 2A,B), with particular emphasis on immune-related genes, such as *CCR4* and *CCL5*, which exhibited significant upregulation in RPS-treated samples (PRJNA1336982).

To ensure the accuracy and reproducibility of our sequencing data, we selected representative DEGs, including fibronectin 1 (*FN1*), *CCR4*, *neuropeptide Y receptor type 2* (NPY2R), *C-X-C motif chemokine ligand 14* (CXCL14), *IL-8*, and *pentraxin* (PTX3) for qPCR validation. The mRNA expression of *FN1*, *CCR4*, and *NPY2R* was significantly upregulated in the RPS + MGAV group compared with the MGAV group (Figure 2C–E), while the mRNA expression of *CXCL14*, *IL8*, and *PTX3* was significantly decreased (Figure 2F–H). These findings not only confirm the reliability of our transcriptomic data, but they also highlight the capacity of RPS to modulate key immune pathways through coordinated gene regulation.

### 3.3. Functional Enrichment Analysis of DEGs and Validation of the CCL5/CCR4 Axis

**Figure 3:**
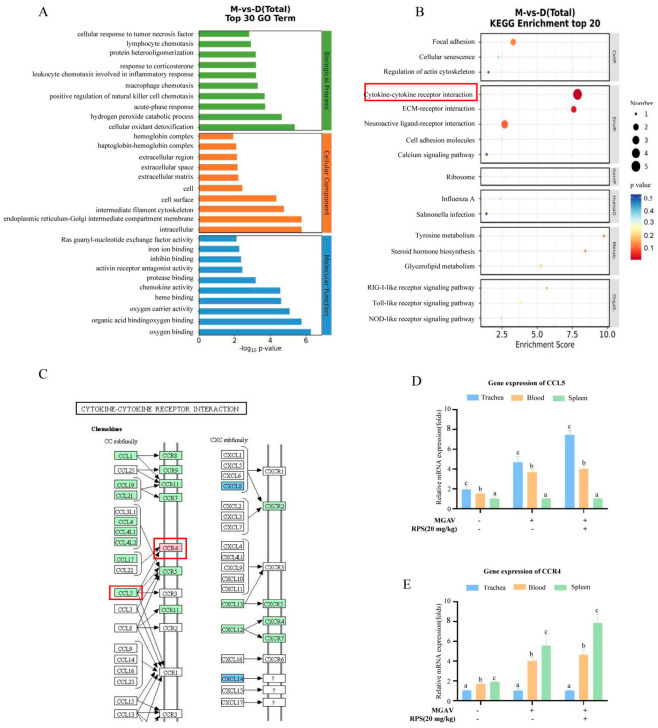
Functional enrichment analysis of DEGs and validation of the CCL5/CCR4 axis. (**A**) GO (top 30 terms) and (**B**) KEGG (top 20 pathways) functional enrichment analysis of DEGs. (**C**) The involvement of DEGs in the cytokine–cytokine receptor interaction pathway, with a highlighted focus on CCL5/CCR4. (**D**,**E**) The mRNA expression of *CCL5* (**D**) and *CCR4* (**E**) in the trachea, blood, and spleen was evaluated by qPCR. In the figure, different lowercase letters indicate significant differences among the groups (*p* < 0.05), while the same letter indicates no significant difference among the groups (*p* < 0.05).

To elucidate the molecular mechanisms underlying RPS-enhanced immunity, we performed bioinformatics analysis of DEGs identified from the spleen transcriptomic data. GO enrichment analysis demonstrated that these DEGs were significantly enriched in critical biological processes, including immune cell migration and chemotaxis, inflammatory response, and redox processes (Figure 3A). KEGG pathway analysis revealed robust enrichment of these genes in key signaling pathways, including cytokine–cytokine receptor interactions, Toll-like receptor signaling pathways, and cell adhesion molecules (Figure 3B). Focusing on the cytokine–cytokine receptor interaction pathway, we identified *CCR4* (encoding a chemokine receptor) as a significantly upregulated gene in RPS-treated samples (Figure 3C). Intriguingly, while *CCR4* expression was markedly enhanced, its cognate ligand *CCL5* did not exhibit statistically significant transcriptional changes in the spleen (*p* < 0.05).

To validate these findings across the tissues, we evaluated the mRNA expression of CCL5 in the trachea, blood, and spleen. Compared with the MGAV alone, RPS co-vaccination synergistically elevated CCL5 mRNA expression in the trachea and blood (Figure 3D), but it was not significantly upregulated in the spleen, which was consistent with the transcriptomic data. Notably, nasal drip immunization using RPS and the MGAV significantly upregulated CCR4 mRNA expression in the trachea, blood, and spleen (Figure 3E). These results collectively confirmed that RPS + MGAV co-administration selectively modulated the CCL5/CCR4 axis, with tissue-specific regulation of *CCL5* expression and systemic upregulation of *CCR4*.

### 3.4. Immunohistochemistry Validation of RPS Treatment-Enhanced CCL5 Protein Expression in Tracheal Tissue

**Figure 4:**
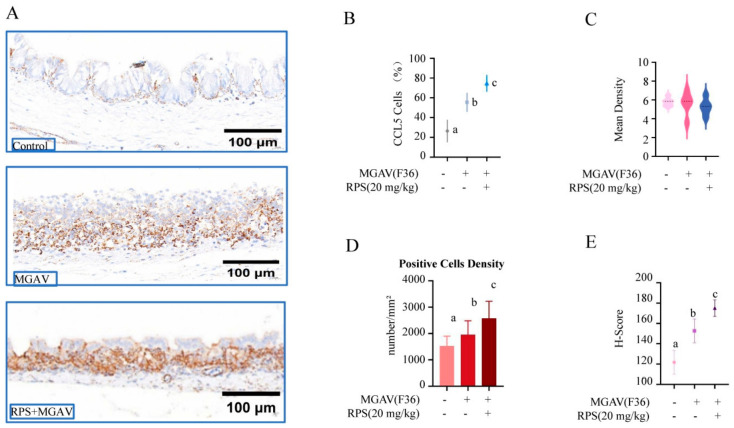
Immunohistochemical validation of CCL5 protein expression in tracheal tissues upon RPS treatment. The tracheal samples from three groups were sectioned and (**A**) stained by immunohistochemistry at 14 days post-primary immunization. (**B**) The percentage of CCL5-positive cells was present and calculated using Aipathwell. (**C**) Mean optical density of CCL5 staining. The average optical density value was calculated as the IOD ÷ area of positive pixels. (**D**) The density of CCL5-positive cells per mm^2^. Positive cell density = number of positive cells ÷ area of the tissue to be tested. (**E**) H-score semi-quantitative assessment. H-score was calculated using the formula ∑ (pi × i), where pi represents the percentage of positive cells corresponding to each grade classification and i denotes the grading scale for positive cells. The grading system was as follows: negative cells without staining scored 0 points; weakly positive cells exhibiting a light-yellow color scored 1 point; moderately positive cells displaying a brownish-yellow hue scored 2 points; and strongly positive cells characterized by a brown coloration scored 3 points. Thus, the H-score can be expressed as (H-score = (percentage of weak intensity cells × 1) + (percentage of moderate intensity cells × 2) + (percentage of strong intensity cells × 3)). In the figure, different lowercase letters indicate significant differences among the groups (*p* < 0.05), while the same letter indicates no significant difference among the groups (*p* < 0.05).

To validate the regulatory effects of RPS on CCL5 expression in vivo, we performed immunohistochemistry analysis of tracheal tissue sections. As demonstrated in Figure 4A, the abundance of CCL5-positive cells was significantly elevated in the RPS + MGAV group compared with both the MGAV group and the control group (*p* < 0.01). This observation was rigorously corroborated by multiple quantitative assessments, including the percentage of CCL5-positive cells (Figure 4B), the mean optical density value of CCL5 staining (Figure 4C), the positive cell density per unit area (Figure 4D), and the H-Score (a composite measure of staining intensity and positive cell proportion) (Figure 4E), which were all significantly increased in the RPS-treated group (*p* < 0.01). These findings collectively demonstrated that RPS combined with the MGAV potently induced CCL5 protein expression in tracheal tissue. These findings provide robust in vivo evidence supporting the immunomodulatory role of RPS through the CCL5 pathway.

### 3.5. RPS Enhanced Macrophage Antigen Phagocytosis and CCL5 Secretion

**Figure 5:**
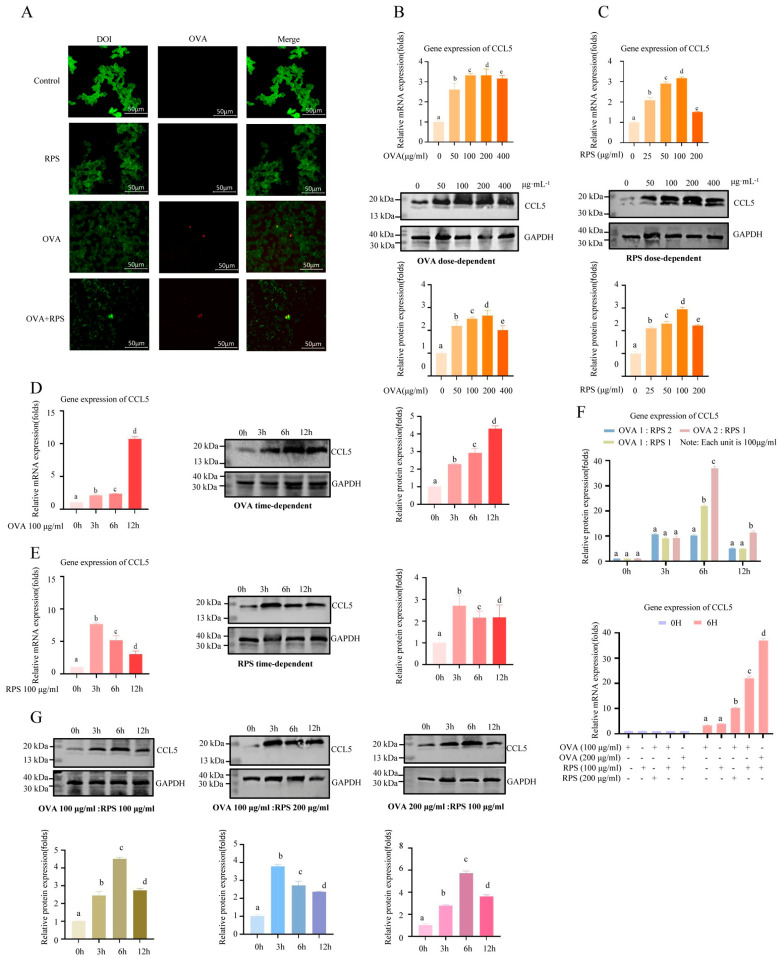
RPS promoted antigen presentation and CCL5 secretion by macrophages. (**A**) RAW264.7 cells were exposed to various concentrations of ovalbumin, RPS, and a combination of ovalbumin and RPS, ranging from 200 μg·mL^−1^ to 100 μg·mL^−1^. Following a 12 h stimulation period, the samples were fixed using paraformaldehyde for subsequent immunofluorescence analysis. Immunofluorescence imaging of ovalbumin phagocytosis by RAW264.7 macrophages was performed (green: ovalbumin; red: cell membrane). (**B**,**C**) To determine the appropriate dosage, the cells were seeded at a density of 5 × 10^5^ cells/well into 6-well plates and cultured until 70–80% confluence. Subsequent to this, a control group lacking any active constituents, an ovalbumin gradient concentration group (50, 100, 200, and 400 μg·mL^−1^), and an RPS gradient concentration group (25, 50, 100, and 200 μg·mL^−1^), with three replicates per group, were utilized. Following a 6 h period of drug treatment at the corresponding concentrations, total RNA and total protein were extracted. qPCR was utilized to measure *CCL5* expression. The housekeeping gene, β-actin, was utilized as the reference, and relative quantification was executed using the 2^−ΔΔCt^ method. To assess CCL5 protein expression, WB analysis was conducted. GAPDH was utilized as the reference protein, and band intensity was quantified using Image J software 1.54p. (**D**,**E**) To determine the most effective treatment time, the cells were seeded at a density of 5 × 10^5^ cells/well into 6-well plates and cultured until 70–80% confluence. Subsequently, the subjects were exposed to either 100 μg·mL^−1^ ovalbumin or 100 μg·mL^−1^ RPS at various timepoints (0, 3, 6, and 12 h). The validation method was identical to that previously described. The 0 h timepoint was designated as the untreated blank control group and functioned as the baseline for the calculation of data from the subsequent timepoints. (**F**,**G**) For combined effect screening, the following combinations were tested: 100 μg·mL^−1^ ovalbumin + 100 μg·mL^−1^ RPS, 100 μg·mL^−1^ ovalbumin + 200 μg·mL^−1^ RPS, and 200 μg·mL^−1^ ovalbumin + 100 μg·mL^−1^ RPS. After treatment, total RNA and total protein were extracted separately. The validation method was the same as above. In the figure, different lowercase letters indicate significant differences among the groups (*p* < 0.05), while the same letter indicates no significant difference among the groups (*p* < 0.05).

To investigate whether RPS modulated CCL5 expression in macrophages, RAW264.7 cells were stimulated using ovalbumin antigen. As shown in Figure 5A, RPS treatment significantly enhanced ovalbumin uptake, as evidenced by the increased fluorescence intensity compared with the control group (*p* < 0.01), demonstrating that RPS promoted antigen phagocytosis in macrophages.

We analyzed the effects of RPS on CCL5 expression at both the transcriptional and translational levels, with emphasis on dose- and time-dependent responses. The results of the dose–response analysis demonstrated that RPS alone resulted in upregulation of *CCL5* mRNA expression in a concentration-dependent manner (Figure 5B,C). It is noteworthy that co-stimulation with ovalbumin and RPS resulted in synergistic enhancement of CCL5 mRNA expression. The most pronounced induction was observed in the group treated with ovalbumin 200 μg·mL^−1^ + RPS 100 μg·mL^−1^ (Figure 5F). Following RPS stimulation, the expression of *CCL5* mRNA reached its maximum at 6 h, while ovalbumin alone induced a peak at 3 h. In both conditions, a decline in mRNA expression was observed, with a duration of 12 h. This transient expression pattern was consistently observed at the protein level (Figure 5D,E). WB analysis further confirmed that co-treatment with RPS and ovalbumin significantly enhanced CCL5 protein expression in a time-dependent manner, with the strongest effect observed in the ovalbumin 200 μg·mL^−1^ + RPS 100 μg·mL^−1^ group (Figure 5G). Together, these results suggest that RPS efficiently promoted CCL5 expression in macrophages through coordinated transcriptional and post-transcriptional mechanisms.

### 3.6. RPS Promoted Lymphocyte Chemotaxis and Macrophage Migration via the CCL5/CCR4 Axis

**Figure 6:**
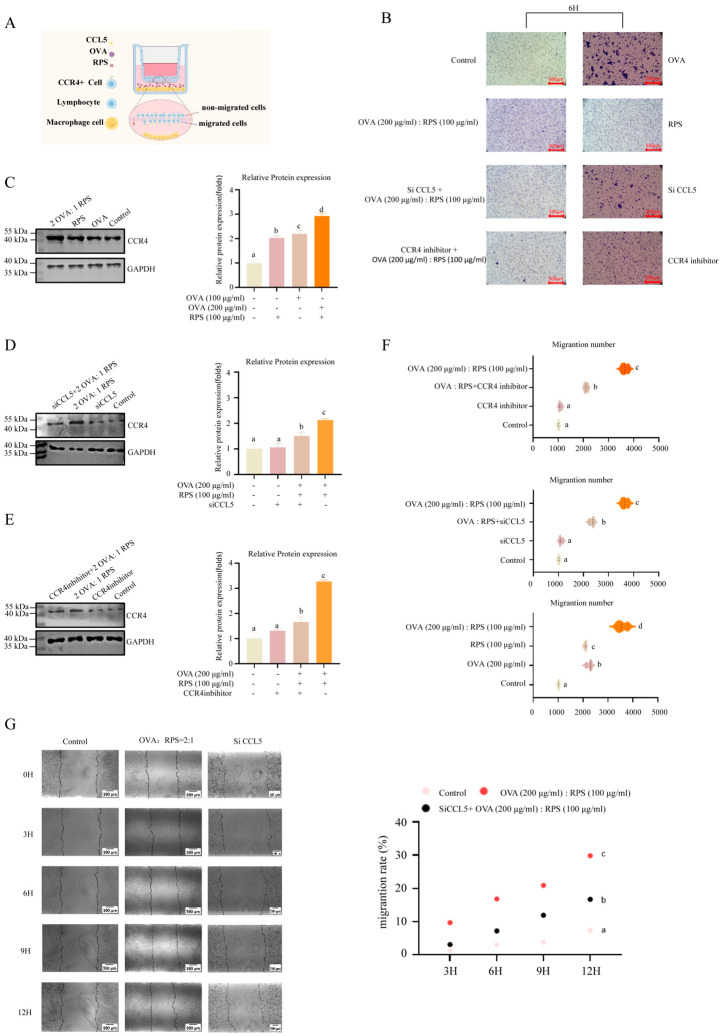
RPS promoted lymphocyte and macrophage chemotaxis and migration via the CCL5/CCR4 axis. Primary lymphocytes were isolated from ICR mice and seeded in the upper chamber of Transwell inserts. RAW264.7 cells were plated in the lower chamber of the Transwell. In the upper chamber, lymphocytes were treated with either PBS or CCR4 inhibitor, but in the lower chamber, RAW264.7 cells were cultured with PBS, 100 μg·mL^−1^ ovalbumin + 200 μg·mL^−1^ RPS, or CCL5 siRNA + 100 μg·mL^−1^ ovalbumin + 200 μg·mL^−1^, separately. Subsequent to a 12 h stimulation period designed to elicit chemotaxis, the migratory cells were analyzed. For the purpose of protein analysis, the lymphocytes that had migrated to the underside of the Transwell membrane were meticulously harvested by scraping the membrane. Total protein was extracted from these cells, and WB was performed to specifically evaluate CCR4 expression within the migrated population. Concurrently, for the purpose of morphological observation and quantification, the membranes from other replicate inserts were fixed using 4% paraformaldehyde and stained using 0.1% crystal violet. The quantity of migrated cells was determined by enumerating stained cells from three random microscopic fields per insert under a light microscope. This combined approach, which entailed the specific analysis of the protein from transmigrated cells in conjunction with direct cell counting, yielded a robust correlation between CCR4 protein expression in the migrated fraction and the quantitative migration data. This approach allowed us to directly correlate the functional migration data with the protein expression profile of the key receptor CCR4 in the migrated cell population. (**A**) Transwell model diagram. (**B**) Representative images of transmigrated lymphocytes. (**C**–**E**) CCR4 protein expression in migrated lymphocytes. (**F**) Statistical analysis of migrated cell numbers. (**G**) RAW264.7 cell chemotaxis was promoted by RPS and ovalbumin. RAW264.7 cells were seeded into 6-well plates with pre-marked reference lines. After 24 h, confluent monolayers were scratched vertically using a sterile pipette tip. Serum-free medium containing the treatment solutions was added. The cells were treated with eight concentrations of PBS, 100 μg·mL^−1^ ovalbumin + 200 μg·mL^−1^ RPS, or CCL5 siRNA + 100 μg·mL^−1^ ovalbumin + 200 μg·mL^−1^ RPS, separately. At 0, 6, 9, and 12 h post-treatment, scratch areas were observed under a microscope and calculated using Image J software 1.54p (National Institutes of Health, Bethesda, MD, USA). All experiments were performed in triplicate. Data are presented as the mean ± SD. Significant differences were determined by one-way ANOVA with Tukey’s post hoc test. In the figure, different lowercase letters indicate significant differences among the groups (*p* < 0.05), while the same letter indicates no significant difference among the groups (*p* < 0.05).

To investigate whether RPS modulated immune cell migration by the CCL5/CCR4 axis, we evaluated lymphocyte chemotaxis toward macrophages using a Transwell co-culture system. The schematic diagram of the Transwell experiment is shown in Figure 6A. As demonstrated in Figure 6B, RAW264.7 cells co-treated with ovalbumin (200 μg·mL^−1^) and RPS (100 μg·mL^−1^) significantly promoted lymphocyte transmembrane migration (*p* < 0.01) compared with the control group. However, this chemotactic effect was substantially attenuated by either silencing CCL5 expression in macrophages (CCL5 siRNA) or blocking the CCR4 receptor on lymphocytes using a CCR4 inhibitor (*p* < 0.01), confirming the pivotal role of the CCL5/CCR4 signaling pathway in mediating this response.

Furthermore, WB analysis (Figure 6C) revealed that CCR4 protein expression increased in lymphocytes following treatment with ovalbumin and RPS, though silencing CCL5 or adding a CCR4 inhibitor suppressed this increase (Figure 6D). Consistent with this finding, quantification of migrated cells (Figure 6E) revealed that the combination of ovalbumin and RPS significantly increased the number of migrated cells to approximately 4000–5000. This increase was reduced to near baseline levels by CCR4 inhibition or CCL5 siRNA treatment. Individual treatment with ovalbumin or RPS alone also induced migration, albeit to a lesser extent than combined treatment (Figure 6F).

To further characterize the impact of RPS on macrophage migratory capacity, we conducted scratch wound-healing assays. As illustrated in Figure 6G, RAW264.7 cells exposed to ovalbumin (200 μg·mL^−1^) and RPS (100 μg·mL^−1^) exhibited significantly accelerated migration rates at 6, 9, and 12 h post-wounding compared with the control group (*p* < 0.01). Notably, CCL5 knockdown resulted in suppression of cell migration, with delayed wound closure observed at all timepoints (*p* < 0.01), underscoring the dependence of RPS-induced macrophage migration on CCL5 signaling.

## 4. Discussion

In this study, we investigated RPS as a candidate vaccine adjuvant and explored its potential immunoregulatory mechanisms. Using a chicken model, we demonstrated that intranasal administration of RPS combined with the MGAV significantly enhanced antibody titers. These findings align with emerging research on plant-derived saponins as a natural vaccine adjuvant. For example, ginsenoside Rb1 boosted IgG and antibody subtypes in the H1N1 influenza vaccine, improved mouse survival post-virus challenge, attenuated lung histopathological damage, and reduced the expression of inflammatory cytokines (*IL-6* and *tumor necrosis factor-alpha*) [25]. In addition, *Radix pseudostellariae* polysaccharide was systematically evaluated and confirmed to be safely administered via the oral or intranasal routes, significantly elevating specific antibody and key cytokine expression levels elicited by the MG-attenuated vaccine [26]. Furthermore, ginseng stem-leaf saponins not only enhance humoral and cellular immune responses to vaccines, such as those used for Newcastle disease and infectious bursal disease, but they also restore vaccine efficacy in models of immunosuppression [27]. Recent studies have revealed that phytosaponins modulate immunity through multiple signaling pathways, including immune organs, immune cells, and cytokines [28]. On the one hand, saponins promote immune organ growth and function; for example, soyasaponins Ab and Bb effectively improve immune status by stimulating the expression of nuclear factor kappa-light-chain-enhancer of activated B cells, *transforming growth factor-beta*, and *interferon-gamma* [28]. On the other hand, they amplify the activity of immune cells; *Panax* but not ginseng saponins increase monocyte–macrophage phagocytosis under immunosuppression, thereby mitigating non-specific immune injury [29], while ginsenoside 20(R)-Rg3 elevates *natural killer* (NK) cell cytotoxicity via upregulation of NKp30, NKp44, NKp46, perforin, and granzyme B [26].

Saponins promote dendritic cell maturation and *IL-12* secretion when co-administered with *lipopolysaccharide*, which further activates *cluster of differentiation*-4 positive T cells and B cells, driving antigen-specific immune responses [30]. Ginseng stem-leaf saponins also improve the activity of intestinal intraepithelial lymphocytes and induce B-cell differentiation into IgA plasma blasts and plasma cells, thereby enhancing mucosal immune defense [31]. Furthermore, astragaloside IV modulates the mitogen-activated protein kinase signaling pathway to partially inhibit macrophage polarization toward the M2 phenotype, consequently limiting cellular invasion, migration, and angiogenesis [6]. Collectively, these findings reveal that saponins enhanced systemic and mucosal immunity through precise regulation of immune cell functions and signaling pathways. Our observation that RPS promoted IL-4 and CCL5 expression in chickens is consistent with these mechanisms [32,33].

Immunohistochemistry analysis confirmed robust CCL5 protein expression in tracheal tissue following RPS treatment. This finding aligns with established mechanisms of chemokine regulation, wherein epithelial cells demonstrate potent CCL5 expression upon exposure to viral mimics (e.g., poly(I:C)) or type-2 cytokines, as evidenced by a study in bronchial epithelial cells [34]. Similarly, macrophages secrete CCL5 in response to pathogen-associated stimuli, such as lipopolysaccharide or *cytosine–phosphate–guanine* oligodeoxynucleotides [35]. These chemokines, in turn, drive the recruitment of lymphocytes and monocytes/macrophages to inflammatory or immune sites. We observed strong CCL5 expression in tracheal tissue, with progressively lower expression in the blood and spleen, suggesting a concentration gradient favoring mucosal sites. This spatial distribution of CCL5 may be because tracheal epithelial and resident macrophages produce CCL5 in response to antigen and RPS stimulation, whereas the systemic circulation and lymphoid organs receive less direct stimulation. Such directed migration of immune cells is particularly critical for respiratory mucosal vaccine strategies, as the respiratory epithelium represents a major barrier that requires sufficient effector cells to reside locally and mount rapid responses.

Transcriptomic analysis revealed that *CCR4* expression was significantly upregulated in the RPS-treated group and enriched in immune cell migration-related pathways. This suggests that RPS may promote the recruitment of immune cells to sites of inflammation or immune activation, an effect partly associated with the CCL5/CCR4 signaling axis. Previous studies have shown that elevated *CCR4* expression on *CCR4-expressing lymphocyte populations* enhances their chemotactic and migratory capacity. For example, in systemic lupus erythematosus, Tregs from healthy individuals exhibited high responsiveness to the CCR4 ligand CCL22, whereas Tregs from patients displayed reduced CCR4 expression and impaired migration [16,36]. CCL5, as one of the key ligands of CCR4, functions as both a T-cell chemoattractant and an immunoregulatory molecule, coordinating leukocyte trafficking and playing a critical role in effective immune responses [37]. CCL5 mediates the recruitment of diverse cytokines, immune cells, and soluble factors to specific sites, thereby participating in immune responses, inflammation, and chemotaxis [38]. Thus, our findings not only highlight a potential mechanistic link between RPS and the CCL5/CCR4 axis as one contributing pathway, but they also provide molecular evidence to explain its adjuvant effects. These results are consistent with a previous report on plant-derived saponins regulating chemokine expression and the dose-dependent promotion of CCL5 secretion by *Rubus chingii* polysaccharide in dendritic cells [39].

RAW264.7 murine macrophages were chosen as the in vitro model because they are a well-characterized cell line for studying phagocytosis and chemokine expression, and because there are no mature commercial chicken macrophage cell lines available. The CCL5/CCR4 axis is highly conserved between mammals and birds, making murine cells a suitable mechanistic proxy. To further verify the mechanism, we conducted in vitro experiments showing that RPS significantly promoted ovalbumin uptake by RAW264.7 macrophages. This suggests that RPS enhances the function of *antigen-presenting cells* (APCs). A previous study showed that saponins interact with membrane cholesterol to facilitate endocytosis in dendritic cells, thereby enabling antigens to access the cytosol, *promoting cross-presentation* (major histocompatibility [MHC] I presentation), while also upregulating MHC II and co-stimulatory molecules to enhance APC function [40]. This finding is also supported by a study on *Radix pseudostellariae* fibrous root saponins, which dose-dependently increased the phagocytic activity and proliferation of RAW264.7 macrophages via the Toll-like receptor 2/4–MyD88–TRIF–nuclear factor-κB signaling pathway [41].

The current study revealed that both RPS alone and in combination with ovalbumin significantly modulated *CCL5* expression in RAW264.7 cells, corroborating that RPS influenced the immune microenvironment through regulation of CCL5 secretion. Importantly, the effects on CCL5 varied depending on the concentration and treatment duration, suggesting a dose- and time-dependent pattern. Similar regulatory dynamics have been reported in recent studies of other plant-derived compounds. For example, ginsenosides and their monomers have been shown to upregulate MHC and co-stimulatory molecules on dendritic cells in a concentration-dependent manner, while ginsenosides Rb3 and Rg3 have been shown to exert dose-dependent modulatory effects on lipopolysaccharide-induced inflammatory responses in RAW264.7 macrophages [42,43]. Moreover, in MG-attenuated vaccine models, oral administration of RPS significantly reduced serum inflammatory factors (IL-1α, CCL4, IL-17A, and IL-6) and dose-dependently restored serum IgA, IgM, and IgG levels in cyclophosphamide-induced immunosuppressed mice, promoting B-cell function recovery [12]. These findings provide important references for optimizing RPS dosage and administration schedules in future studies.

The Transwell migration and scratch assays confirmed that RPS promoted the migration of CCR4-positive cells and macrophages, consistent with the classic role of the CCL5/CCR4 axis in T-cell trafficking. For instance, recent studies have demonstrated that local expression or exogenous delivery of CCL5 markedly enhanced effector T-cell migration into tissues and boosted local interferon-γ-mediated anti-tumor activity [44,45]. Another previous study showed that CCL5 can elicit cell-type-specific signaling responses by binding to distinct *G protein-coupled receptors*, including CCR1, CCR3, CCR4, and CCR5 [46]. In our chicken model, RPS enhanced localized immune competence through two complementary mechanisms: strengthening antigen presentation and promoting the recruitment of CCR4-positive effector and regulatory cells to the trachea. This dual action could explain the robust immune response observed alongside reduced tracheal inflammation. This study focuses on immunogenicity enhancement rather than direct vaccine efficacy. We did not assess protection against *Mycoplasma gallisepticum* challenge, including MG colonization load, clinical signs, or pathological lesions. Therefore, we refrain from claiming vaccine efficacy. Future challenge studies are needed to evaluate the protective effect of RPS as an adjuvant.

## 5. Conclusions

**Figure A1:**
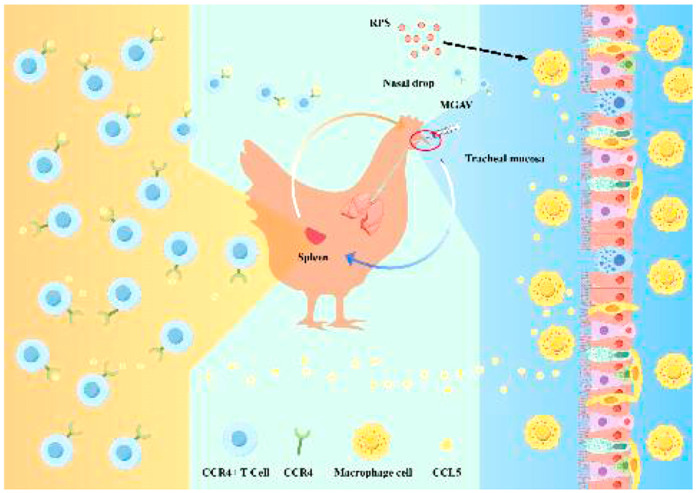
Molecular mechanism model. Schematic diagram illustrating the proposed mechanism by which RPS promotes immunocyte migration and chemotaxis via the CCL5/CCR4 signaling axis.

In conclusion, this study demonstrated that RPS enhanced immune responses to the MG vaccine by regulating the CCL5/CCR4 signaling axis. This promoted the activation, chemotaxis, and antigen-presenting capabilities of immune cells, particularly macrophages and CCR4-positive cells. These findings shed new light on the adjuvant mechanism of RPS and support its potential application in conjunction with the MG vaccine and others (Figure A1).

## Data Availability

The data that support the findings of this study are available from the corresponding author upon reasonable request.

## Appendix A

**Table A1:** Primer information.

Genes	Sequences	Size/bp
β-Actin (Chicken)	F:5′-CCAAAGCCAACAGAGAGAAGAT-3′	120
	R:5′-CATCACCAGAGTCCATCACAAT-3′	
IL-4 (Chicken)	F:5′-CTCACTGCCCACCCTGCTG-3′	129
	R:5′-ACCTCTCCCTGGATGTCATTCAC-3′	
CCL5 (Chicken)	F:5′-GCTGTGTCCCTCTCCATCCTC-3′	94
	R:5′-AGTTGAAGCAGCACACGGTTG-3′	
FN1 (Chicken)	F:5′-AACCTGCCTGATACTGCTACCTC-3′	99
	R:5′-ACTCTCCTGATGTTCCTCCACTG-3′	
CCR4 (Chicken)	F:5′-CCTGGTCATTGTGGTCCTCT-3′	101
	R:5′-TCCCACTGTAGAACCCAACC-3′	
NPY2R (Chicken)	F:5′-GCCTTCCAGCTAGTCAGTGATATTG-3′	149
	R:5′-GCCGTCCTGTAGTTGTTATTCATCC-3′	
IL-8 (Chicken)	F:5′-ACCTCACTGCAAGAATGTGGAA-3′	80
	R:5′-GAAGCTGAATGGCGTTGTCT-3′	
PTX3 (Chicken)	F:5′-CCAAGAGAAGAACGGCTGCTGTG-3′	129
	R:5′-TCTCCGCCCTGTGCTGCTATC-3′	
CXCL14 (Chicken)	F:5′-GTGACCCTGTGGACGAAAGTGAG-3′	113
	R:5′-ACCCTGCCCTTCTCCTTCCATAC-3′	
β-Actin (mouse)	F:5′-GAGACCTTCAACACCCCAGCC-3′	264
	R:5′-AATGTCACGCACGATTTCCC-3′	
CCL5 (mouse)	F:5′-GTATTTCTACACCAGCAGCAGCAAG-3′	120
	R:5′-TCTTGAACCCACTTCTTCTCTG-3′	
Si-CCL5	5′-UCUUGAUUCUGACCCUGUAUATT,-3′	
	5′-UAUACAGGGUCAGAAUCAAGATT-3′	
